# Trainees’ self-reported challenges in knowledge translation practice and research

**DOI:** 10.1186/1472-6963-14-S2-P129

**Published:** 2014-07-07

**Authors:** Robin Urquhart, Evelyn Cornelissen, Shalini Lal, Kristine Newman, Dwayne Van Eerd, Byron Powell, Vivian Chan

**Affiliations:** 1 Department of Surgery, Dalhousie University, Halifax, Nova Scotia, Canada; 2 Department of Family Practice, University of British Columbia, Vancouver, British Columbia, Canada; 3 Department of Psychiatry, McGill University, Montreal, Quebec, Canada; 4 Daphne Cockwell School of Nursing, Ryerson University, Toronto, Ontario, Canada; 5 Institute for Work & Health, Toronto, Ontario, Canada; 6 Brown School, Washington University in St. Louis, St. Louis, Missouri, USA; 7 Vancouver Coastal Health, Vancouver, British Columbia, Canada

## Background

Knowledge translation (KT) refers to the process of moving knowledge into healthcare practice and policy. The practice of KT is about helping decision-makers become aware of knowledge and facilitating their use of it in their day-to-day work. The science of KT is about studying the determinants of knowledge use and investigating strategies to support the adoption, implementation, and sustained use of knowledge in healthcare practice and policy. An increasing number of trainees are developing careers in KT practice and/or KT research. Given the infancy of this field, there may be unique challenges that trainees face as they develop their careers in KT. This paper is one of two from a study about KT trainees’ perspectives on KT research and practice. The purpose of this paper was to identify challenges that KT trainees face in their KT practice or research.

## Materials and methods

The study population was a convenience sample of trainees associated with the KT Trainee Collaborative (KTTC) and/or the KT Canada Summer Institutes (KTSI). Trainees affiliated with the KTTC and KTSI were emailed a link to a survey through FluidSurveysTM. Descriptive statistics (e.g., frequencies) were calculated for trainee demographics. Thematic analysis was used to analyze open-ended response data related to the following question: What are the major challenges you face in KT practice or KT research? Three investigators independently coded and categorized the data, then met to collaboratively identify, merge, and refine themes.

## Results

The survey response rate was 62% (44/71) but only 49% (35/71) responded to the challenges question. 51% (18/35) of these respondents reported doing both KT practice and research; 43% (15/35) doing KT research only; and 6% (2/35) doing KT practice only. Trainees identified six major challenges related to their KT work: KT is not recognized as a distinct field of practice or study; colleagues’ limited understanding of KT practice and/or research; competing priorities and limited time (particularly to undertake KT practice); a lack of KT-specific resources (e.g., funding, training opportunities, peers); difficulty collaborating and communicating across sectors and cultures; and the difficulty inherent in investigating KT (e.g., designing and testing multilevel interventions, challenges with adaptation).

## Conclusions

The findings suggest that KT trainees experience specific challenges in their work, many of which arise because of an underdeveloped understanding of KT; limited structures/infrastructure to support individuals undertaking KT; and the inherently interdisciplinary nature of KT and the resultant complexities in scientific inquiry in this field.

